# An Experimental Approach for Investigating Freezing of Gait in Parkinson’s Disease Using Virtual Reality and Neural Sensing: A Pilot Study

**DOI:** 10.3390/s25134036

**Published:** 2025-06-28

**Authors:** Mandy Miller Koop, Anson B. Rosenfeldt, Kathryn Scelina, Logan Scelina, Colin Waltz, Andrew S. Bazyk, Visar Berki, Kyle Baker, Julio N. Reyes Torres, Enio Kuvliev, Sean Nagel, Benjamin L. Walter, James Liao, David Escobar, Kenneth B. Baker, Jay L. Alberts

**Affiliations:** 1 Department of Biomedical Engineering, Cleveland Clinic, Cleveland, OH 44195, USA; koopm@ccf.org (M.M.K.); rosenfa2@ccf.org (A.B.R.); scelink@ccf.org (K.S.); scelinl@ccf.org (L.S.); waltzc@ccf.org (C.W.); bazyka2@ccf.org (A.S.B.); berkiv@ccf.org (V.B.); 2 Department of Neurosciences, Cleveland Clinic, Cleveland, OH 44195, USA; bakerk14@ccf.org (K.B.); reyestj@ccf.org (J.N.R.T.); kuvliee@ccf.org (E.K.); escobad2@ccf.org (D.E.); bakerk6@ccf.org (K.B.B.); 3 Center for Neurological Restoration, Cleveland Clinic, Cleveland, OH 44195, USA; nagels@ccf.org (S.N.); walterb7@ccf.org (B.L.W.); liaoj2@ccf.org (J.L.)

**Keywords:** Parkinson’s disease, freezing of gait, deep brain stimulation, electroencephalography, virtual reality

## Abstract

Freezing of gait (FOG) is a disabling symptom associated with Parkinson’s disease (PD). Its understanding and effective treatment is compromised due to the difficulty in reliably triggering FOG in clinical and laboratory environments. The Cleveland Clinic-Virtual Home Environment (CC-VHE) platform was developed to address the challenges of eliciting FOG by combining an omnidirectional treadmill with immersive virtual reality (VR) environments to induce FOG under physical, emotional, and cognitive triggers. Recent developments in deep brain stimulation devices that sense neural signals from the subthalamic nucleus in real time offer the potential to understand the underlying neural mechanism(s) of FOG. This manuscript presents the coupling of the CC-VHE technology, VR paradigms, and the experimental and analytical methods for recording and analyzing synchronous cortical, subcortical, and kinematic data as an approach to begin to understand the nuanced neural pathology associated with FOG. To evaluate the utility and feasibility of coupling VR and neural sensing technology, initial data from one participant are included.

## 1. Introduction

Falls occur in nearly 80% of individuals with Parkinson’s disease (PD), are the largest motor-related contributor to health care costs and often catalyze the transition to dependent care settings [1,2]. Freezing of gait (FOG) and postural instability and gait difficulties (PIGD) are well-recognized precursors to PD-related falls. Freezing of gait is often described by individuals with PD as a feeling that their feet are glued to the floor [3]. Although FOG can emerge at any point in the disease process, the frequency and duration of episodes typically increase as the disease progresses and can result in injuries and hospitalizations [4]. Despite the prevalence and substantial impact on quality of life, the pathophysiological mechanisms underlying FOG are unclear, and effective treatments remain elusive as traditional therapies have yielded inconsistent results—leading some to suggest that FOG may be treatment refractory [5,6].

Deep brain stimulation (DBS) has been used to effectively manage PD symptoms such as tremor and bradykinesia for over 25 years; however, it is not recognized as a robust, effective treatment for FOG [7]. Recent advances in DBS technology offer a novel avenue to examine the neural mechanisms underlying FOG in individuals with PD. The Medtronic Percept™ system, approved by the FDA in 2020, combines stimulation with novel sensing capabilities to monitor and record neural activity (i.e., local field potentials (LFPs)) at the stimulation site in real time [8,9]. The novel sensing capabilities of the Percept system may provide an avenue for investigating the pathological activity in the basal ganglia during FOG, a crucial factor in identifying the neural underpinnings of FOG.

A critical challenge in evaluating FOG is that it is often episodic and unpredictable, and triggers for FOG are multifactorial and vary between individuals [10,11]. Common triggers for FOG include walking through doorways, turning, initiating gait, coupling gait with a secondary attention-demanding task (e.g., walking while talking), and navigating stressful or anxiety-provoking situations [12]. These triggers have led to the development of multiple theoretical models of FOG, including the threshold, cognitive, and interference models. Briefly, the threshold model hypothesizes FOG occurs when the accumulation of various motor deficits compound to a point of motor breakdown [13]. The cognitive model highlights PD executive dysfunction and hypothesizes that the failure to process response-conflict results in faster—but less congruent—response-decisions, resulting in FOG [14]. Finally, the interference model suggests that disordered neuronal crosstalk between motor, cognitive, and limbic circuits overloads the information-processing capacity of the basal ganglia and its depleted dopamine-producing neurons, resulting in FOG [15]. While these models provide theoretical guidance in understanding FOG, they have not been systematically evaluated or verified with neural data from the basal ganglia or basal ganglia-thalamocortical circuitry. A fundamental limitation in understanding the neural mechanisms of FOG and developing effective treatments is the difficulty in reliably triggering episodes in laboratory [16,17] or clinical environments [18,19]. Pairing virtual reality (VR) with an omnidirectional treadmill to replicate real-world environments known to trigger FOG offers a promising approach. Previous studies have demonstrated the feasibility of leveraging immersive VR environments with treadmill-based tasks for assessments of individuals with PD, both with and without FOG [12,20]. However, to date, no project has combined immersive technology and neural sensing to evaluate patterns of neural activity across the cortex and basal ganglia under FOG triggered by unique stimuli. Combining these novel technologies can transform the understanding and treatment of FOG by transitioning the field from viewing FOG and PIGD as monoliths to having a more nuanced understanding of PD-related lower extremity dysfunction. A detailed understanding of patient-specific declines in FOG or PIGD will enhance treatment.

The Cleveland Clinic-Virtual Home Environment (CC-VHE) platform was developed to elicit FOG in individuals with PD with various environmental stimuli that operationalize the theoretical models of FOG: (1) Physical (threshold model), (2) Anxiety (interference model), and (3) Cognitive (cognitive model). This manuscript explores the feasibility of the CC-VHE platform as a standardized method of triggering FOG events and a roadmap for collecting, processing, and interpreting cortical (EEG) and STN (LFP) data during locomotion. Data from one individual with PD and a history of FOG are presented to evaluate the feasibility of the experimental methods and their capability to induce FOG, and the effectiveness in recording and analyzing synchronous neural and kinematic data. This experimental paradigm provides a novel approach to test the theoretical models of FOG while identifying the neurophysiological effects of various FOG triggers. The CC-VHE and methods presented provide a cornerstone for deriving new insights into the neural underpinnings of FOG that may, with further implementation and data, facilitate more effective, patient-specific treatment.

## 2. Materials and Methods

### 2.1. Equipment and Data Collection Procedures

**Figure 1:**
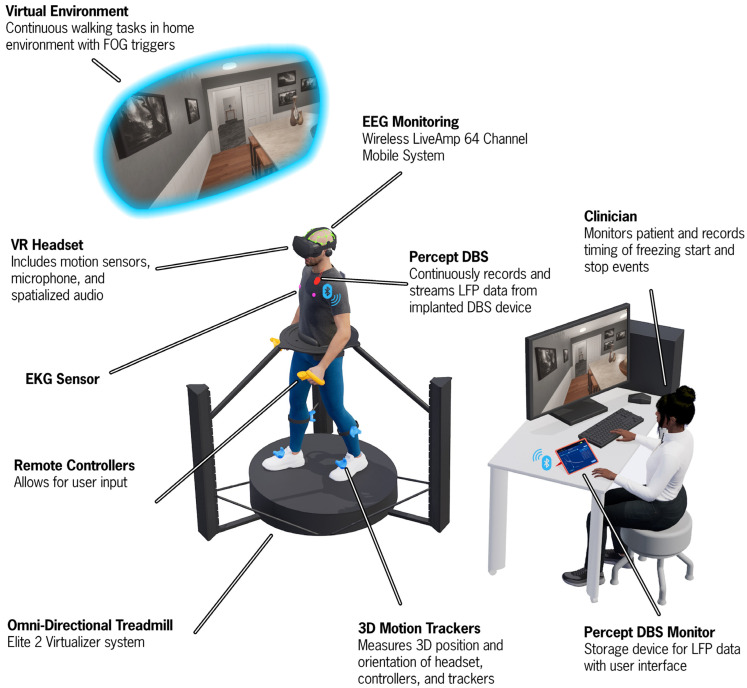
An illustration of the Cleveland Clinic-Virtual Home Environment (CC-VHE) hardware and software components. The participant walks and turns on an omnidirectional treadmill to navigate three virtual home environment scenarios that operationalize the theoretical models of FOG. Synchronized local field potential, electroencephalogram, kinematic, and electrocardiogram data are simultaneously recorded while a clinician notes the onset and offset of freezing of gait episodes.

The CC-VHE platform (Figure 1) combines an omnidirectional treadmill with immersive VR-based scenarios designed to replicate real-world conditions to reliably and safely elicit FOG episodes in individuals with PD. Its development was based on our VR grocery shopping task used to objectively quantify instrumental activities of daily living in older adults and individuals with PD [20,21,22].

#### 2.1.1. CC-VHE VR Hardware

The CC-VHE consists of four components: (1) an omnidirectional treadmill (Cyberith Virtualizer Elite 2, Niederosterreich, Austria), (2) a VR headset (Valve Index, Bellevue, WA, USA), (3) four lower extremity VR trackers with integrated Inertial Measurement Units (IMUs) (HTC VIVE Tracker 3.0, Taoyuan City, Taiwan), and (4) two (i.e., left and right) hand controllers (Valve Index, Bellevue, WA, USA).

The omnidirectional treadmill is a self-paced motion system platform that allows the user to locomote (walking, turning, stopping) in any direction while being secured in a waist-worn harness to prevent falls. User motion is tracked through six optical motion sensors in the baseplate and an optical rotation sensor in the ring around the waist of the user. The VR headset delivers fully immersive digital content with a refresh rate of 120 Hz. Four VR trackers are secured on the participant’s feet and shanks to provide biomechanical data while the participant physically locomotes through the virtual environment. Data from the VR trackers is sampled at 60 Hz. Hand controllers were used by the participant to interact with the virtual environment and indicate responses in the Cognitive module. The treadmill, headset, and hand controllers all work in concert to orient the participant and ensure synchronization of the visual display with the participant’s physical movement, hence minimizing incidence of VR sickness. The ultra-high framerate of 1000 Hz from the optical sensors that monitor the waist and foot motion guarantee fast and precise response and motion tracking.

#### 2.1.2. CC-VHE Modules

The CC-VHE has three assessment modules designed to operationalize three theoretical models of FOG: (1) Physical (threshold model), (2) Anxiety (interference model), and (3) Cognitive (cognitive model). A virtual home-kitchen environment, which includes a kitchen island (6 m × 1 m), is the beginning and ending location for each module. Prior to the assessment modules, the participant completes a Reference module, where they complete one lap of single-task walking around the kitchen island at a self-selected walking speed without distraction.

**Figure 2:**
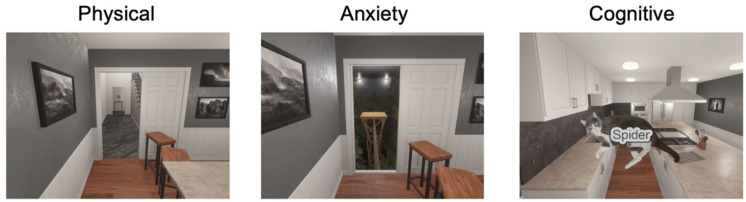
First-person views of the CC-VHE assessment modules that include the following: (1) The Physical module includes narrow doorways and changes in flooring patterns; (2) The Anxiety module requires the navigation of elevated, narrow walkways over a forest of trees; and (3) The Cognitive module requires walking while performing a simultaneous cognitive-motor task identifying congruent or incongruent stimuli. The example illustrated in this figure represents an incongruent stimulus: the word “Spider” is incongruent with the picture of a cat; the correct response from the participant would be to activate the hand controller in the left hand.

Figure 2 provides a first-person view of the three CC-VHE modules. The virtual kitchen is a 4 × 12 m room with a large kitchen island spanning and double doors at the end of the island. Participants begin each module in the home-kitchen area of the environment. In the *Physical module*, the participant walks from the starting point in the kitchen toward the double doors. When the participant is within 1.6 m of the double doors, they automatically open to reveal a hallway. Once through the double doors, the participant walks down the hallway, completes a turn to another narrower hallway, and they return to the starting position. Throughout the Physical module, the participant encounters alterations in the environment known to trigger FOG: change in flooring patterns, narrow doorways (~68 cm), narrow hallways (~70 cm), and multiple 90-degree turns. In the *Anxiety module*, the participant walks toward the double doors from the common starting position. The doors open when they are within 1.6 m and reveal a narrow walkway (~0.10 m) suspended 8 m above the ground. The participant crosses the anxiety-producing elevated plank (6 m long) to a platform (2 m × 2 m), turns 180 degrees, and then returns over the plank and back to the original starting position. Finally, the *Cognitive module* consists of a cognitive-motor dual task in which the participant continuously walks around the kitchen island while simultaneously performing a picture interference task (i.e., identify whether the picture of the animal is congruent or incongruent with the text by pressing the trigger buttons on the right and left hand controllers, respectively, at 1.5 s intervals) [23]. The Cognitive module is 120 s in duration. The Physical and Anxiety modules’ durations vary depending on navigation speed.

#### 2.1.3. CC-VHE Kinematic Recordings

Three-dimensional linear and angular positions of the feet, shank, head, and waist are direct outputs of the CC-VHE system and obtained via IMU sensors from the foot and shank trackers, the headset, and an optical rotation sensor in the treadmill that monitors the orientation of the participant’s waist.

#### 2.1.4. Electroencephalogram (EEG) Recordings

EEG signals are acquired using the Brain Vision LiveAmp 64 mobile system electrodes (Brain Vision actiCAP slim; Brain Vision, Garner, NC, USA). EEG data are recorded at a sampling rate of 500 Hz from 64 channels through ActiCap Slim active Ag/AgCl electrodes, referenced to an electrode placed at the FCz position, embedded in an elastic cap with electrode locations conforming to the international 10-10 system [24]. Electrode cables are secured with cable ties and kept close to the head to minimize sway during recording. EEG data are recorded with the Brain Vision Recorder 1.26.0001 software, and impedances are restricted to ~5–10 kOhm.

#### 2.1.5. Subthalamic Nucleus (STN)-LFP Recordings

Bilateral STN-LFP data are continuously recorded at 250 Hz using the Percept PC system (Medtronic, Galway, Ireland) in the Indefinite Streaming Mode. Data from 6 channel pairs, three per hemisphere, LFP_0–2_, LFP_0–3_, LFP_1–3_- for each STN, are streamed wirelessly and stored on a tablet (Galaxy Tab S5e; Samsung, Suwon-si, Republic of Korea) paired to the Percept PC system. The LFP data are passed through multiple filters on the device prior to exporting. All data are passed through two low-pass filters at 100 Hz, and two high-pass filters at 1 Hz [25]. The filtered LFP magnitudes (µV) in the time domain are downloaded as a JSON file for offline analysis in MATLAB (R2024a).

#### 2.1.6. Transcutaneous Electrical Nerve Stimulation (TENS) Device

A commercially available TENS device (BioStim Plus; BioMedical Life Systems Inc., Carlsbad, CA, USA) is utilized to inject an electrical artifact into the LFP and EEG data streams for synchronization purposes. Specifically, two adhesive disc surface electrodes (Natus Inc., Middleton, WI, USA) are placed on the participant, one near the extension cable insertion point on the implantable pulse generator (IPG) and the other over the contralateral mastoid process. Multiple 0.5 s TENS bursts (250 µs, 80 Hz, 1.5 mA) are injected into the neural data streams.

#### 2.1.7. Video Recordings

All trials are filmed for post hoc review.

### 2.2. Data Alignment

Data alignment between the neural data from the DBS and EEG devices to gait events is achieved by injecting electrical artifacts from the following systems: (1) a Raspberry Pi and (2) a TENS device. A control computer sends a signal to a host Raspberry Pi to signal the VR and video recording to initiate and terminate data collection for each module, and the timing of these events is recorded by the Brain Vision Recorder software (EEG system) (Sync A). The Raspberry Pi also sends synchronization pulses (Sync A) at 15 s intervals to the VR and Brain Vision Recorder systems during each module to detect any time delays between the two systems over the course of a trial. Prior to starting each module, a TENS device is utilized to inject a detectable electrical artifact (multiple 0.5 s on/0.5 s off electrical bursts (250 µs, 80 Hz, and 1.5 mA pulses)) into both the LFP and EEG data streams (Sync B), providing a link between the LFP data and the other systems. Throughout the entire testing session, the Brain Vision Recorder software continuously collects data and serves as the data collection hub, as it captures both the Sync A and Sync B pulses utilized to temporally align the data streams.

Post hoc data alignment is performed in MATLAB. For Sync A, the start of the VR trial is aligned to the first Sync pulse A in the EEG data. For Sync B, the EEG and LFP data are band-pass filtered with a cut-off frequency centered around the TENS pulse frequency (80 Hz) (fourth-order, pass band with cut-off frequencies of 60 and 100 Hz), and then a cross-correlation analysis (MATLAB xcorr function) that minimizes the error between the TENS artifact recorded in both data streams is used to remove the lag between the filtered-EEG and -LFP data streams. After the EEG and LFP data streams are aligned, the EEG and LFP data are segmented into individual trials aligned with the VR and video data streams.

**Figure 3:**
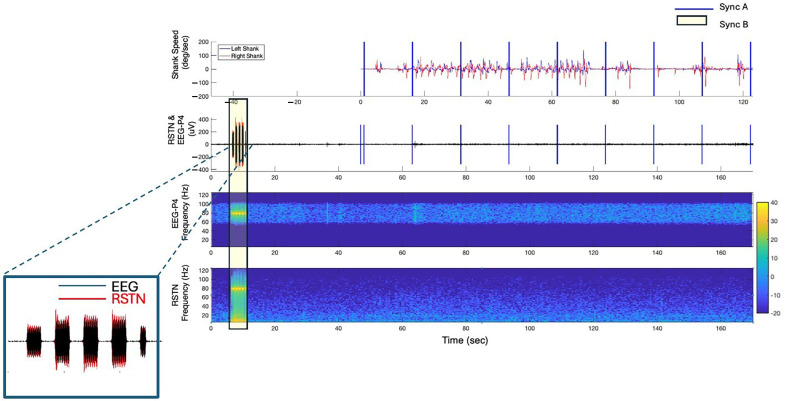
Temporally aligned kinematic, EEG and LFP data from a representative trial in the off–Off condition. Two devices were utilized to inject artifacts into the data streams to facilitate temporal alignment between the EEG, LFP and the kinematic data: the Raspberry Pi (Sync A) and the TENS (Sync B). The insert illustrates the alignment between the EEG and LFP data streams.

The error in synchronization is quantified using the timing of the synchronization pulses (Sync A and Sync B) in the temporally aligned VR, EEG and LFP data streams. For each trial, the mean value of the absolute difference in timing of the Sync A pulses between the VR and EEG data were determined. For Sync B, the amount of error was quantified using the band-pass filtered EEG and LFP data to identify the timepoints of the rising and falling edges of each burst (see Figure 3 insert). The first and last bursts were removed from the analysis as only bursts with full amplitudes were utilized to quantify the alignment. The mean value of the absolute time differences between the two data streams of the rising and falling points for each burst were calculated and utilized to quantify the temporal error between the LFP and EEG data streams (Sync B). The sum of the errors determined in Sync A and B was utilized as the final measurement of error for each trial.

### 2.3. Identification of Freezing and Walking Epochs

#### 2.3.1. Visual Identification of FOG Episodes

FOG episodes are initially identified in real time by a physical therapist (Board-Certified Clinical Specialist in Neurologic Physical Therapy; AR) during the testing session. To record the timing of FOG episodes, the VR data recording program is designed to receive inputs from the keyboard and record the timing of the FOG episode onset and offset in the VR data file and send timing events to the Brain Vision Recorder Software. Keyboard data are time-locked with the VR data streams and sampled at 60 Hz. Following the testing session, all identified FOG episodes are reviewed by the physical therapist (AR) utilizing the synchronized video recordings. Once FOG episodes are confirmed or refined as needed, a custom MATLAB script is used to create a text file with the finalized onset and offset of timestamps of each FOG episode that is utilized in all subsequent analyses.

#### 2.3.2. Visual Identification of Walking Epochs (Walk)

The same physical therapist reviewed video from the Reference module and the three assessment modules prior to stimuli introduction and identified epochs when the participant was single-task walking in a straight line without FOG, referred to as “Walk”. The same custom MATLAB script described for the FOG episodes was used to record the onset and offset of the Walk time points. The video-reviewed Walk epochs were utilized as a baseline condition. Specifically, the Walk epochs were used to characterize non-freezing behavior, from which neural outcomes from the LFP and EEG data were extracted and compared to EEG and LFP outcomes during FOG.

### 2.4. Neural Data Analysis and Outcomes

#### 2.4.1. LFP Data Analysis and Outcomes

For each trial, the LFP data in the JSON files were uploaded to MATLAB where the adjacent bipolar channels were reconstructed from the LFP data extracted in the JSON file, resulting in six channels per hemisphere; extracted: LFP_0–3_, LFP_0–2_, LFP_1–3_, reconstructed: LFP_0–1_ = LFP_0–3_ − LFP_1–3_, LFP_1–2_ = LFP_1–3_ + LFP_0–2_ − LFP_0–3_, LFP_2–3_ = LFP_0–3_ − LFP_0–2_. Prior to analysis, all data were visually inspected for movement artifacts and any epochs with artifacts were removed from the dataset.

#### 2.4.2. Selection of LFP Data Channels for Analysis

Using the LFP data form the Reference module in session one (off meds—Off DBS condition), the power spectral densities (PSDs) for each of the 12 channels are calculated using the Welch method (pWelch MATLAB function) with a 1 s Hanning window and 0.5 s overlap [26]. A generalized power law (1/f ^alpha^) is applied to each channel’s PSD to simulate the physiological baseline activity in the brain. Average power in the beta band (13–35 Hz) is extracted from the PSD of the entire trial. The channel with the largest beta power (i.e., average power between 13 and 35 Hz above the 1/f baseline) per hemisphere, right (R) and left (L), is utilized for subsequent analysis across all modules and henceforth referred to as RSTN-LFP and LSTN-LFP.

#### 2.4.3. EEG Data Analysis and Outcomes

EEG data are analyzed with the EEGLAB toolbox in MATLAB. The preprocessing steps include the removal of line noise (60 Hz) using the cleanline algorithm, resampling data to 250 Hz, and bandpass filtering (cut-off frequencies of 1 and 41 Hz). The EEG channel data are then visualized for artifacts and identified noisy time segments are manually removed before processing with the Automated Subspace Reconstruction (ASR) [27] plug-in for EEGLAB (“FlatlineCriterion” = 5, “ChannelCriterion” = 7, “BurstCriterion” threshold = 10 in standard deviation units). For EEG artifact removal, Independent Component Analysis (ICA) and a labeling method to decompose the EEG data into spatially independent components (ICs) are applied before automatically removing noise components and reconstructing the EEG signals [28,29]. ICs labeled as channel noise, line noise, muscle artifact, or eye artifact with a probability of 70% or more are removed from the source signals. The entire dataset is then re-referenced to the common average.

#### 2.4.4. Selection of EEG Channel Utilized for Analysis

Previous studies have identified cortical areas involved in FOG and include the supplementary motor area (Fz), primary motor area (Cz), navigational movement area (P4), and the primary visual receiving area (O1) [30]. For the case study presented in this manuscript, the P4 channel, recognized as the navigational movement area, demonstrated the greatest change between FOG and Walk epochs and is presented in the results.

#### 2.4.5. Calculation of LFP and EEG Outcome Measures

For each module, the video-reviewed onset and offset of the FOG episodes and Walk epochs that were identified by the clinician are utilized to segment the EEG and LFP (RSTN and LSTN) time-series data into FOG and Walk epochs. Next, epochs with a minimum duration of 2 s are included in the analysis. For each epoch with a duration > 2 s, the data are segmented into windows 2.0 s in duration using a moving window with a 0.5 s time step. For each 2.0 s segment, PSDs are calculated using the Welch method (pWelch MATLAB function) with a 1 s Hanning window and 0.5 s overlap [26], and average power values for each 1 Hz frequency band are calculated. PSDs are calculated for each 2 s window of FOG episodes and Walk epochs for the LFP and EEG data and are used in the statistical analysis.

### 2.5. Statistical Analysis

Monte Carlo permutation tests are used to determine statistical difference between the amplitude of oscillatory activity (i.e., power) in LFP and EEG data during FOG and Walk epochs for each module. For this analysis, the actual difference in average power between FOG and Walk epochs for each 1 Hz frequency band between 8 and 35 Hz for STN-LFP and 1 and 35 Hz for EEG is compared to the difference in average power calculated between two groups selected at random from a shuffled dataset that includes both FOG and Walk power values. This permutation is completed ten thousand times. For each 1 Hz frequency band, if the actual difference in the FOG and Walk average power values is outside the 95% confidence limit of the mean difference of the shuffled data, this difference is considered significant. The False Discovery Rate (fdr_bh.m) is used to account for multiple comparisons (i.e., 28 frequency bands, 8–35 Hz) for STN-LFP (35 frequency bands, 1–35 Hz) for EEG with an alpha of 0.05. For the case study presented in this manuscript, a secondary analysis was performed in which isolated, significant frequency bands, i.e., 1 Hz bands in which the power values were significantly different between FOG and Walk but were surrounded by non-significant bands, were removed. This was performed to provide a conservative estimate of the differences between FOG and Walk epochs, given that the results are based on a single participant.

## 3. Proof-of-Concept Results

Data from a single-participant case study are presented to evaluate the feasibility of the data collection paradigm and the data analysis methods and to provide initial neural data for the generation and refinement of hypotheses related to the possibility that the stimuli producing the FOG episode may produce different patterns of cortical and subcortical neural activity.

The participant was a 65-year-old male who had been diagnosed with PD 6 years prior. He had a history of FOG and reported two falls over the previous 6 months. His levodopa equivalent daily dose (LEDD) was 1150 mg/day, and he underwent bilateral STN DBS surgery approximately eight months prior to data collection. Testing was completed in two sessions on a single day: (1) off medication–Off DBS, and (2) on medication–Off DBS. The off-antiparkinsonian medication state was operationally defined as 12+ hours since the last medication dose. The day prior to testing, the participant was assessed on medication–On DBS. The MDS UPDRS-III assessments were performed by a clinician blinded to medication and DBS status, and scores were: 47 on–On, 51 on–Off and 59 off–Off states. This study was approved by the Cleveland Clinic Institutional Review Board, and the participant provided informed consent.

### 3.1. All CC-VHE Modules Elicited FOG Episodes

The CC-VHE successfully elicited FOG in all three modules for both medication conditions. In the off–Off condition, the participant experienced 10 FOG episodes (Physical module n = 4, Anxiety module n = 2, Cognitive module n = 4) for a total duration of ~80 s. The Cognitive module produced the longest duration of FOG episodes (four episodes, totaling 40.1 s), approximately two-fold greater than either the Physical or Anxiety modules. The participant provided correct responses to 29 of the 79 picture-animal name items presented in the Cognitive module evenly distributed throughout the 120 s trial.

The participant also experienced FOG episodes in all the modules in the on medication–Off DBS condition, and medication reduced the number and duration of FOG episodes by approximately 60% (Table 1).

### 3.2. Synchronization Across All Data Streams

Alignment between the EEG and kinematic data was quantified as the difference in the time stamps of the synchronization pulses (Figure 3—Sync A) that were sent at 15 s intervals across all three modules. The average absolute error in temporal alignment between the EEG and kinematics data streams was 3.4 (SD = ±2.9) ms. Next, the temporal alignment accuracy between the LFP and EEG data streams was determined by detecting the average difference in the duration of the TENS bursts (Figure 3—Sync B) in the two data streams. On average, the error in the temporal alignment was less than 20.7 (SD = ±21.5) ms for Sync B pulses. The total alignment error across all data streams was 24.2 (SD = ±19.9) ms.

### 3.3. Beta Power Was Reduced with Medications

**Figure 4:**
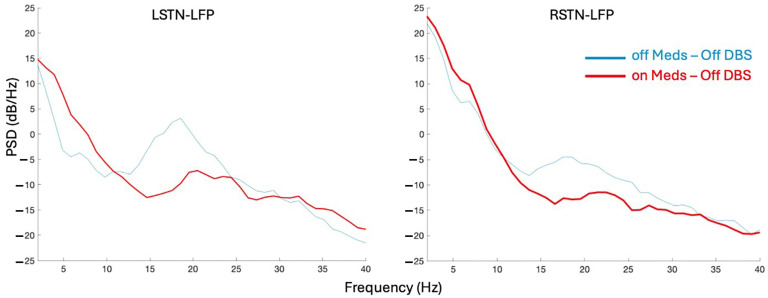
Power spectral densities of local field potential data from the left and right subthalamic nucleus (STN) in both medication states.

Bilateral STN-LFP recordings from a representative module, Cognitive, were compared between the off–Off and on–Off states (Figure 4). Calculation of the average power in the beta band (13–35 Hz) across the 120 s trial showed that the STN beta power was greater in the off–Off condition than on–Off by 48% and 40% for the LSTN and RSTN, respectively.

### 3.4. Neural Synchrony Increased During FOG

**Figure 5:**
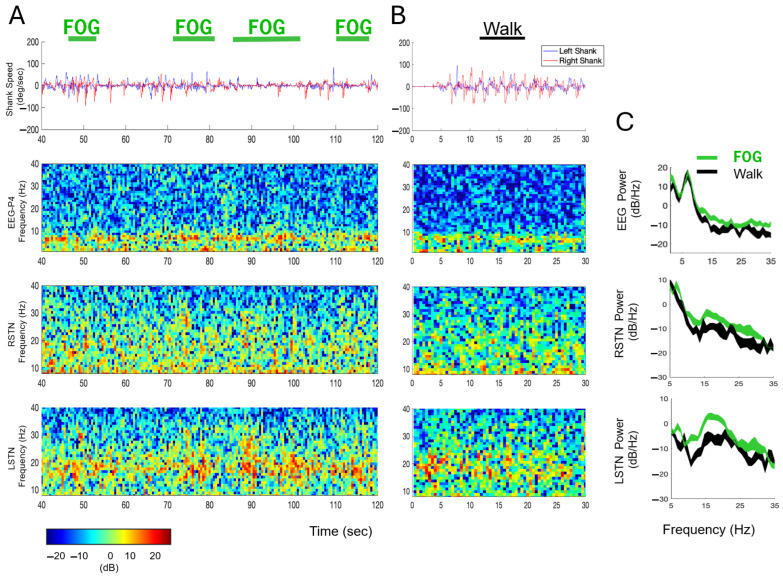
Kinematic, EEG, and LFP data with FOG episodes and Walk epochs in the off–Off condition. (**A**) Top-to-bottom: Shank angular speed, EEG, and right and left STN LFP data during a sample, 80 s (40–120 s) period from the Cognitive module. Four FOG epochs are identified with green horizontal bars. The EEG and STN activity spectrograms are color-coded according to the key presented below. (**B**) Shank angular speed, EEG, and STN data from 30 s of the Reference module, with a Walk epoch identified with a black horizontal bar. (**C**) Power spectral densities of the EEG and LFP data during all FOG (green traces) and Walk epochs (black traces) during the Cognitive module. The width of the green and black traces in the PSDs represents the 95% confidence intervals of the mean.

Temporally aligned kinematic, EEG, and LFP data from representative trials in the off–Off condition are presented in Figure 5. Specifically, Figure 5A shows data from the Cognitive module with four clinician-identified FOG epochs. Figure 5B presents data from the Reference module. Visual inspection of the kinematic data supports the accuracy of the clinician’s classification of FOG and Walk epochs, as the shank angular speed is slower during the FOG epochs and faster and more regular during the Walk epoch. The EEG and STN-LFP data from these epochs are illustrated in spectrograms which demonstrate subtle spectral changes over time between the FOG and Walk epochs. Welch’s PSD estimates were calculated across all FOG episodes during the Cognitive module (FOG total time = 40.1 s; Figure 5C—green traces) and all Walk epochs averaged across all modules (Walk total time = 24.4 s) to measure differences in neural activity between FOG (Figure 5C—green traces) and Walk epochs (Figure 5C—black traces). Visual inspection of the PSDs show elevated power (i.e., increased neural synchrony [31]) during the FOG epochs compared to Walking in cortical (P4 EEG electrode—navigational movement area) and subcortical (L- and R-STN) regions for nearly all beta-band frequencies (13–35 Hz).

### 3.5. Changes in STN-LFP Between FOG and Walking Across CC-VHE Modules

**Figure 6:**
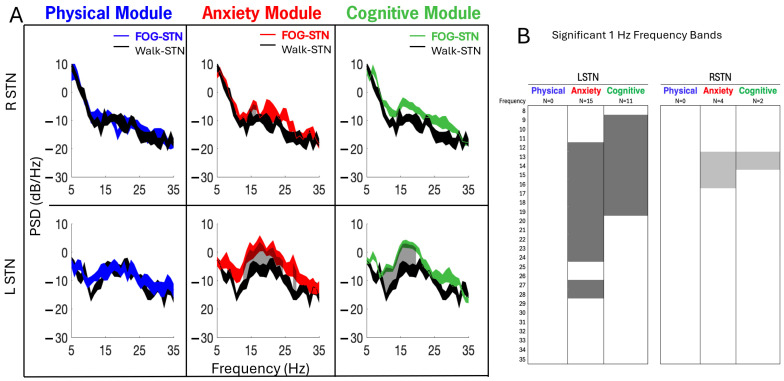
(**A**) In the example participant, power spectral densities of local field potentials from the left (LSTN) and right (RSTN) subthalamic nucleus showed elevated PSDs during FOG (blue, red, and green traces) compared to Walk epochs (single-task, straight-line walking epochs averaged across all modules, black trace) for Anxiety and Cognitive modules; left > right. Gray shaded areas (panels **A** and **B**) represent the 1 Hz frequency bands between 8 and 35 Hz that were significantly increased (*p* < 0.05, FDR correction) during FOG compared to Walk. In (**A**), the width of the PSD traces represents the 95% confidence interval of the mean. (**B**) depicts the number (N) and range of 1 Hz frequencies that were significantly elevated during FOG compared to Walk per module.

Power spectral densities of STN-LFP data recorded in the off–Off condition showed increased PSDs (i.e., increased neural synchrony) during FOG (color traces) compared to Walk epochs across all three modules (Figure 6A). Gray shaded areas represent frequencies, 1 Hz bands between 8 and 35 Hz, that were significantly different between FOG and Walk epochs (*p* < 0.05, FDR correction). Figure 6B depicts the differences in the number (N) and the ranges of the elevated 1 Hz frequency across all modules. Compared to Walk epochs, power in the LSTN was elevated across more frequencies than the RSTN during FOG (26 versus 6 significantly elevated 1 Hz bands, respectively). Specifically, power was elevated in the LSTN in the Anxiety (15 bands) and Cognitive (11 bands) modules in the alpha-low beta frequencies (8–20 Hz). For both the RSTN and LSTN, the Anxiety module displayed the largest number of 1 Hz frequency bands that were elevated during FOG and the largest range of elevated frequencies; there were no significant changes in power during FOG compared to Walk in the Physical modules. The Anxiety module was the only module where high beta frequencies (>20 Hz) were significantly different during FOG compared to Walk, and only for the LSTN.

### 3.6. Changes in EEG Between FOG and Walking Across CC-VHE Modules

**Figure 7:**
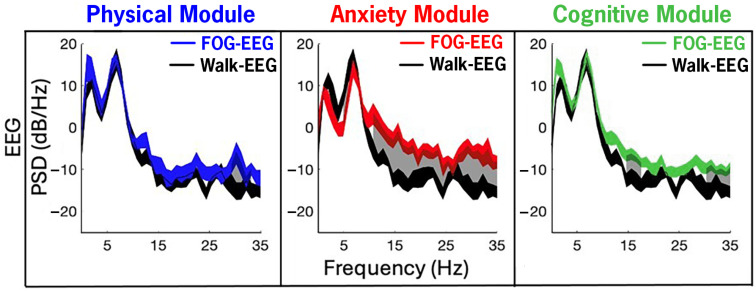
Cortical EEG (channel P4—navigational movement area) power in the navigational movement during FOG was elevated compared to Walk epochs, with the largest differences in the Anxiety and Cognitive modules. Neural synchrony was increased during FOG (color traces) compared to Walk (single-task, straight-line walking epochs averaged across all modules, black trace) in the Anxiety and Cognitive modules. Gray shaded areas represent the 1 Hz frequency bands between 1 and 35 Hz that were significantly increased (*p* < 0.05, FDR correction) during FOG compared to Walk. The width of the traces in the PSDs represents the 95% confidence intervals of the mean.

Figure 7 illustrates EEG PSDs from the P4 channel recorded from all therapy during the CC-VHE modules and, consistent with the LFP data, shows increased PSD (i.e., increased neural synchrony) during FOG epochs compared to Walk epochs. Significant differences in power between FOG and Walk were detected in 14%, 80%, and 29% of all 1 Hz frequency bands (1–35 Hz) in the Physical, Anxiety, and Cognitive modules, respectively.

## 4. Discussion

The results from the case report support the CC-VHE platform as a feasible framework for eliciting FOG, while simultaneously gathering behavioral and neural data to provide insight into the neural underpinnings of PD-related FOG. Utilizing the experimental methods presented, the EEG, LFP, kinematic, and video data were successfully synchronized and segmented into FOG and epochs. The participant experienced 14 FOG episodes with a cumulative duration of 110 s, during ~12 min of active data collection. The participant experienced at least one FOG episode in each module and in each therapeutic state (i.e., off–Off and on–Off), providing an opportunity to compare the neural activity during FOG between the two conditions. Furthermore, the expected improvements when on medication, reflected in the reduced number and duration of FOG episodes and the decrease in LFP beta power, lend support to the integrity of the subcortical neuronal data recorded and the validity of the analysis methods presented. In sum, the results support the feasibility of utilizing the CC-VHE and the described experimental and analytical methods for further investigation into the neural signature of FOG and provide promising, albeit initial, data supporting potential neural differences in FOG based on the type of stimuli triggering the episode.

Reliably eliciting FOG in clinical and research settings is a well-recognized challenge in the treatment of FOG in individuals with PD. FOG occurs in a variety of situations during daily activities, such as navigating in crowded spaces under stressful conditions or while performing a second, discrete task (i.e., dual-tasking)—scenarios and environments that are often difficult to replicate in a clinical setting. As a result, many FOG studies have used physical obstacles, such as doorways or office dividers, to create obstacle courses with narrow corridors in exam rooms and hallways to elicit FOG episodes in clinical and laboratory settings [32,33,34]. These paradigms leave unanswered questions regarding the role of cognitive and limbic inputs in the underlying mechanisms of FOG as they utilize primarily physical triggers. Virtual reality expands FOG assessment beyond the controlled clinical environment to more real-world activities, like navigating around one’s home. Notably, previous PD studies utilizing VR and mixed reality have been successful at triggering FOG [35,36,37]; however, many have primarily used non-immersive VR and assessed FOG by foot taps on a pedal with participants in a seated position, or via stepping in place paradigms as a surrogate for ambulatory FOG assessments [38]. Additionally, these paradigms often expose participants to incongruent visual and proprioceptive feedback which can cause VR sickness. The use of an omnidirectional treadmill in the CC-VHE mitigates motion sickness by synchronizing visual and vestibular input [21]. Of note, the participant in this case report successfully completed all testing modules in a single session, despite the option to complete over multiple days. After completing each module, the participant was questioned regarding any discomfort, or if they were experiencing any signs of VR sickness (nausea, dizziness, headache, eye strain, etc.). At no time did they endorse symptoms of VR sickness. Ultimately, the CC-VRS platform brings real-world conditions, known to induce FOG, to research and clinical environments, which has been elusive until now. Coupled with recent advancements in EEG and LFP technology, this experimental paradigm provides a potential framework for characterization of the neurophysiological underpinnings of FOG to investigate unanswered questions regarding the variability of neural activity in FOG across various triggers.

The CC-VHE’s incorporation of different FOG-provoking modules may facilitate opportunities to answer recent inquiries into the causal role of cognitive and limbic inputs in the underlying neural mechanisms contributing to FOG. While conclusions cannot be generalized from a single case report, the current data provide support and rationale for testing the hypothesis that not all FOG episodes are the same from a neural signature perspective. Rather, unique signatures may be linked to the environmental stimuli that are contributing to the triggering of the FOG event. Larger studies are planned using the developed methods to test this hypothesis.

To date, the reported changes in neural activity recorded in the cortex and the STN that are associated with FOG are inconsistent. Cortical recordings during FOG have demonstrated increases in theta activity [39] in the central region during freezing and increases in alpha band power during FOG compared to walking in the central and occipital areas [30]. Studies have also shown that for turn-triggered FOG, beta and theta power are enlarged predominantly in the occipital and parietal areas [40,41,42]. Previous studies suggest the extent of beta band attenuation may be related to the severity of the FOG episode, where high and low beta power may be modulated differently in the cortex during the transition period from normal walking to freezing compared to during a FOG episode [43]. In addition, the prominent finding across several STN-LFP studies is an increase in beta activity in the STN during FOG compared to walking [44,45,46]. The experimental paradigm creates a roadmap to systematically evaluate the cortical-subcortical signal during walking and FOG.

The STN is a subcortical nucleus that is approximately 12 × 5 × 3 mm^3^ in size, located in the basal ganglia, recognized as a key nucleus is motor control, and is the primary target for DBS for treatment of PD. Early somatotopic mappings of the STN illuminated segregated pathways for processing of emotional, cognitive, and motor information that project to striatum and motor cortex through direct and indirect pathways. Previous FOG studies have used known triggers that can be arranged into three broad categories of conditions that result in FOG: alterations in the physical environment, adding anxiety-provoking stimuli to the environment, and incorporating dual-task conditions [47]. These triggers have led to the development of multiple theoretical models of FOG, including threshold, cognitive, and interference models. While these models provide theoretical guidance, to the best of our knowledge, no study to date has attempted to characterize neural activity in the STN associated with FOG across these domains. Based on the segregated pathways in the STN, the FOG neural signature in the STN-LFP and cortical recordings may be unique to the specific type of trigger.

The STN-LFP and cortical data and initial data presented demonstrate the feasibility of collecting these behavioral and neural data to further understand the underpinnings of FOG in people with PD under virtual conditions that replicate real-world environments known to cause freezing. It is well established that FOG is a largely patient-specific phenomenon, with individual differences in frequency, severity, and triggers of FOG. Given this heterogeneity, using the CC-VHE and the described experimental methods, on-going analyses of data from a larger investigation aims to expand the limited understanding of the cortical and subcortical activity associated with FOG and provides an approach testing previous and emerging theoretical models of FOG.

As part of the larger CC-VHE study, additional metrics and analyses, including beta burst, peak power, connectivity measures between the LFP and EEG data sets across all 12 LFP channels (six per hemisphere) and 64 EEG channels will be utilized to characterize the patient- and trigger-specific variability in neural activity associated with FOG and may reveal more universal signatures of FOG. Using the study design and analysis methods presented here, this larger study seeks to expand the current methods for eliciting FOG episodes to elucidate the neural underpinnings of this largely refractory symptom.

## 5. Conclusions

In conclusion, the purpose of this project was to test the feasibility of using the CC-VHE, coupled with the experimental paradigm and data analysis approach, to characterize the neurophysiology associated with FOG across unique environmental stimuli. The use of VR, coupled with a wireless LFP recording system and mobile EEG, reflects a novel approach to study and understand the neural mechanism(s) of FOG in ecologically valid, real-world environments, while retaining the critical ability to manipulate specific FOG triggers and retain experimental control. Results from the case study provides rationale to evaluate the hypothesis that the neurophysiology of FOG may be related to its environmental triggers. A larger sample size, using the CC-VHE and methods detailed previously, aims to systematically test this hypothesis. Greater insight into the potential relationship between the environment and neural activity will facilitate more effective treatment interventions that leverage advances in adaptive closed-loop DBS, transcranial magnetic stimulation (TMS), or transcranial direct current stimulation (tDCS).

## Figures and Tables

**Table 1:** Number and duration of FOG episodes and percentage of time spent in FOG episodes during each CC-VHE module.

	Off Medication–Off DBS			On Medication–Off DBS		
	Physical	Anxiety	Cognitive	Physical	Anxiety	Cognitive
Number of FOGs (count)	4	2	4	2	1	1
Duration of FOGs (s)	21.5	18.5	40.1	11.8	10.6	7.6
Percent of trial in FOG	12.8%	10.7%	33.6%	6.1%	7.4%	6.3%

## Data Availability

Research data will be made available upon reasonable request to the corresponding author.

## References

[1] Bjornestad A., Tysnes O.B., Larsen J.P., Alves G. (2016). Loss of independence in early Parkinson disease: A 5-year population-based incident cohort study. Neurology.

[2] Jasinska-Myga B., Heckman M.G., Wider C., Putzke J.D., Wszolek Z.K., Uitti R.J. (2012). Loss of ability to work and ability to live independently in Parkinson’s disease. Parkinsonism Relat. Disord..

[3] Schaafsma J.D., Balash Y., Gurevich T., Bartels A.L., Hausdorff J.M., Giladi N. (2003). Characterization of freezing of gait subtypes and the response of each to levodopa in Parkinson’s disease. Eur. J. Neurol..

[4] Lieberman A., Deep A., Olson M.C., Smith Hussain V., Frames C.W., McCauley M., Lockhart T.E. (2019). Falls When Standing, Falls When Walking: Different Mechanisms, Different Outcomes in Parkinson Disease. Cureus.

[5] Palakurthi B., Burugupally S.P. (2019). Postural Instability in Parkinson’s Disease: A Review. Brain Sci..

[6] Cao S.S., Yuan X.Z., Wang S.H., Taximaimaiti R., Wang X.P. (2020). Transverse Strips Instead of Wearable Laser Lights Alleviate the Sequence Effect Toward a Destination in Parkinson’s Disease Patients with Freezing of Gait. Front. Neurol..

[7] Weiss D., Schoellmann A., Fox M.D., Bohnen N.I., Factor S.A., Nieuwboer A., Hallett M., Lewis S.J.G. (2020). Freezing of gait: Understanding the complexity of an enigmatic phenomenon. Brain.

[8] Jimenez-Shahed J. (2021). Device profile of the percept PC deep brain stimulation system for the treatment of Parkinson’s disease and related disorders. Expert. Rev. Med. Devices.

[9] van Rheede J.J., Feldmann L.K., Busch J.L., Fleming J.E., Mathiopoulou V., Denison T., Sharott A., Kuhn A.A. (2022). Diurnal modulation of subthalamic beta oscillatory power in Parkinson’s disease patients during deep brain stimulation. NPJ Park. Dis..

[10] Zhang L.L., Zhang L., Dong J., Zhao Y., Wang X.P. (2022). Factors Contributing to Malnutrition in Parkinson’s Disease Patients with Freezing of Gait. Front. Neurol..

[11] Banks S.J., Bayram E., Shan G., LaBelle D.R., Bluett B. (2019). Non-motor predictors of freezing of gait in Parkinson’s disease. Gait Posture.

[12] Bekkers E.M.J., Mirelman A., Alcock L., Rochester L., Nieuwhof F., Bloem B.R., Pelosin E., Avanzino L., Cereatti A., Della Croce U. (2020). Do Patients with Parkinson’s Disease with Freezing of Gait Respond Differently Than Those Without to Treadmill Training Augmented by Virtual Reality?. Neurorehabil. Neural Repair..

[13] Plotnik M., Giladi N., Hausdorff J.M. (2012). Is freezing of gait in Parkinson’s disease a result of multiple gait impairments? Implications for treatment. Parkinsons Dis..

[14] Vandenbossche J., Deroost N., Soetens E., Coomans D., Spildooren J., Vercruysse S., Nieuwboer A., Kerckhofs E. (2012). Freezing of gait in Parkinson’s disease: Disturbances in automaticity and control. Front. Hum. Neurosci..

[15] Lewis S.J., Barker R.A. (2009). A pathophysiological model of freezing of gait in Parkinson’s disease. Parkinsonism Relat. Disord..

[16] Chen C.C., Yeh C.H., Chan H.L., Chang Y.J., Tu P.H., Yeh C.H., Lu C.S., Fischer P., Tinkhauser G., Tan H. (2019). Subthalamic nucleus oscillations correlate with vulnerability to freezing of gait in patients with Parkinson’s disease. Neurobiol. Dis..

[17] Ehgoetz Martens K.A., Hall J.M., Georgiades M.J., Gilat M., Walton C.C., Matar E., Lewis S.J.G., Shine J.M. (2018). The functional network signature of heterogeneity in freezing of gait. Brain.

[18] Bloem B.R., Hausdorff J.M., Visser J.E., Giladi N. (2004). Falls and freezing of gait in Parkinson’s disease: A review of two interconnected, episodic phenomena. Mov. Disord..

[19] Okuma Y., Silva de Lima A.L., Fukae J., Bloem B.R., Snijders A.H. (2018). A prospective study of falls in relation to freezing of gait and response fluctuations in Parkinson’s disease. Park. Relat. Disord..

[20] Rosenfeldt A.B., Waltz C., Zimmerman E., Davidson S., Hastilow K., Alberts J.L. (2024). An immersive virtual reality shopping task detects declines in instrumental activities of daily living in individuals with Parkinson’s disease. Park. Relat. Disord..

[21] Alberts J.L., McGrath M., Miller Koop M., Waltz C., Scelina L., Scelina K., Rosenfeldt A.B. (2022). The Immersive Cleveland Clinic Virtual Reality Shopping Platform for the Assessment of Instrumental Activities of Daily Living. J. Vis. Exp..

[22] Lewis M.M., Waltz C., Scelina K., Scelina L., Owen K., Hastilow K., Koop M.M., Rosenfeldt A.B., Alberts J.L. (2023). Older adults exhibit declines in instrumental activities of daily living during a virtual grocery shopping task. Front. Virtual Real..

[23] Cochrane B.A., Pratt J. (2022). The item-specific proportion congruency effect transfers to non-category members based on broad visual similarity. Psychon. Bull. Rev..

[24] Jasper H.H. (1958). The ten-twenty electrode system of the International Federation. Electroencephalogr. Clin. Neurophysiol..

[25] Medtronic BrainSense™ Whitepaper Update for A610 v3.0. https://www.medtronicacademy.com/en-us/document/brainsense--whitepaper-update-for-a610-v3-0/DBS.

[26] Welch P. (1967). The use of fast Fourier transform for the estimation of power spectra: A method based on time averaging over short, modified periodograms. IEEE Trans. Audio Electroacoust..

[27] Mullen T., Kothe C., Chi Y.M., Ojeda A., Kerth T., Makeig S., Cauwenberghs G., Jung T.P. (2013). Real-time modeling and 3D visualization of source dynamics and connectivity using wearable EEG. Annu. Int. Conf. IEEE Eng. Med. Biol. Soc..

[28] Delorme A., Palmer J., Onton J., Oostenveld R., Makeig S. (2012). Independent EEG sources are dipolar. PLoS ONE.

[29] Delorme A., Makeig S. (2004). EEGLAB: An open source toolbox for analysis of single-trial EEG dynamics including independent component analysis. J. Neurosci. Methods.

[30] Cao Z., John A.R., Chen H.T., Martens K.E., Georgiades M., Gilat M., Nguyen H.T., Lewis S.J.G., Lin C.T. (2021). Identification of EEG Dynamics During Freezing of Gait and Voluntary Stopping in Patients with Parkinson’s Disease. IEEE Trans. Neural Syst. Rehabil. Eng..

[31] Buzsaki G., Draguhn A. (2004). Neuronal oscillations in cortical networks. Science.

[32] Snijders A.H., Nijkrake M.J., Bakker M., Munneke M., Wind C., Bloem B.R. (2008). Clinimetrics of freezing of gait. Mov. Disord..

[33] Nieuwboer A., Dom R., De Weerdt W., Desloovere K., Fieuws S., Broens-Kaucsik E. (2001). Abnormalities of the spatiotemporal characteristics of gait at the onset of freezing in Parkinson’s disease. Mov. Disord..

[34] O’Day J., Syrkin-Nikolau J., Anidi C., Kidzinski L., Delp S., Bronte-Stewart H. (2020). The turning and barrier course reveals gait parameters for detecting freezing of gait and measuring the efficacy of deep brain stimulation. PLoS ONE.

[35] Gilat M., Shine J.M., Bolitho S.J., Matar E., Kamsma Y.P., Naismith S.L., Lewis S.J. (2013). Variability of Stepping during a Virtual Reality Paradigm in Parkinson’s Disease Patients with and without Freezing of Gait. PLoS ONE.

[36] Killane I., Fearon C., Newman L., McDonnell C., Waechter S.M., Sons K., Lynch T., Reilly R.B. (2015). Dual Motor-Cognitive Virtual Reality Training Impacts Dual-Task Performance in Freezing of Gait. IEEE J. Biomed. Health Inform..

[37] Janssen S., de Ruyter van Steveninck J., Salim H.S., Cockx H.M., Bloem B.R., Heida T., van Wezel R.J.A. (2020). The Effects of Augmented Reality Visual Cues on Turning in Place in Parkinson’s Disease Patients with Freezing of Gait. Front. Neurol..

[38] Zhao H., Feng Z., Hao S., Tan H., Zhan S., Liu W., Lu Y., Cao C. A Virtual Reality (VR) based Comprehensive Freezing of Gait (FOG) Neuro-electrophysiologic Evaluation System for People with Parkinson’s Disease (PD). Proceedings of the 2023 45th Annual International Conference of the IEEE Engineering in Medicine & Biology Society (EMBC).

[39] Shine J.M., Handojoseno A.M., Nguyen T.N., Tran Y., Naismith S.L., Nguyen H., Lewis S.J. (2014). Abnormal patterns of theta frequency oscillations during the temporal evolution of freezing of gait in Parkinson’s disease. Clin. Neurophysiol..

[40] Handojoseno A.M., Gilat M., Ly Q.T., Chamtie H., Shine J.M., Nguyen T.N., Tran Y., Lewis S.J., Nguyen H.T. (2015). An EEG study of turning freeze in Parkinson’s disease patients: The alteration of brain dynamic on the motor and visual cortex. Annu. Int. Conf. IEEE Eng. Med. Biol. Soc..

[41] Quynh Tran L., Ardi Handojoseno A.M., Gilat M., Rifai C., Ehgoetz Martens K.A., Georgiades M., Naik G.R., Tran Y., Lewis S.J.G., Nguyen H.T. (2017). Detection of turning freeze in Parkinson’s disease based on S-transform decomposition of EEG signals. Annu. Int. Conf. IEEE Eng. Med. Biol. Soc..

[42] Asher E.E., Plotnik M., Gunther M., Moshel S., Levy O., Havlin S., Kantelhardt J.W., Bartsch R.P. (2021). Connectivity of EEG synchronization networks increases for Parkinson’s disease patients with freezing of gait. Commun. Biol..

[43] Karimi F., Niu J., Gouweleeuw K., Almeida Q., Jiang N. (2021). Movement-related EEG signatures associated with freezing of gait in Parkinson’s disease: An integrative analysis. Brain Commun..

[44] Yin Z., Zhu G., Liu Y., Zhao B., Liu D., Bai Y., Zhang Q., Shi L., Feng T., Yang A. (2022). Cortical phase-amplitude coupling is key to the occurrence and treatment of freezing of gait. Brain.

[45] Singh A. (2018). Oscillatory activity in the cortico-basal ganglia-thalamic neural circuits in Parkinson’s disease. Eur. J. Neurosci..

[46] Toledo J.B., Lopez-Azcarate J., Garcia-Garcia D., Guridi J., Valencia M., Artieda J., Obeso J., Alegre M., Rodriguez-Oroz M. (2014). High beta activity in the subthalamic nucleus and freezing of gait in Parkinson’s disease. Neurobiol. Dis..

[47] Conde C.I., Lang C., Baumann C.R., Easthope C.A., Taylor W.R., Ravi D.K. (2023). Triggers for freezing of gait in individuals with Parkinson’s disease: A systematic review. Front. Neurol..

